# Supplementary material for: Taspase1 Facilitates Topoisomerase IIβ-Mediated DNA Double-Strand Breaks Driving Estrogen-Induced Transcription
Source: Cells. 2023 Jan 18;12(3):363. doi: 10.3390/cells12030363 (PMC9913075; doi:10.3390/cells12030363)
Supplement: Supplementary file 1 [file cells-12-00363-s001.zip › cells-2088563-supplementary.pdf]

Supplementary Materials

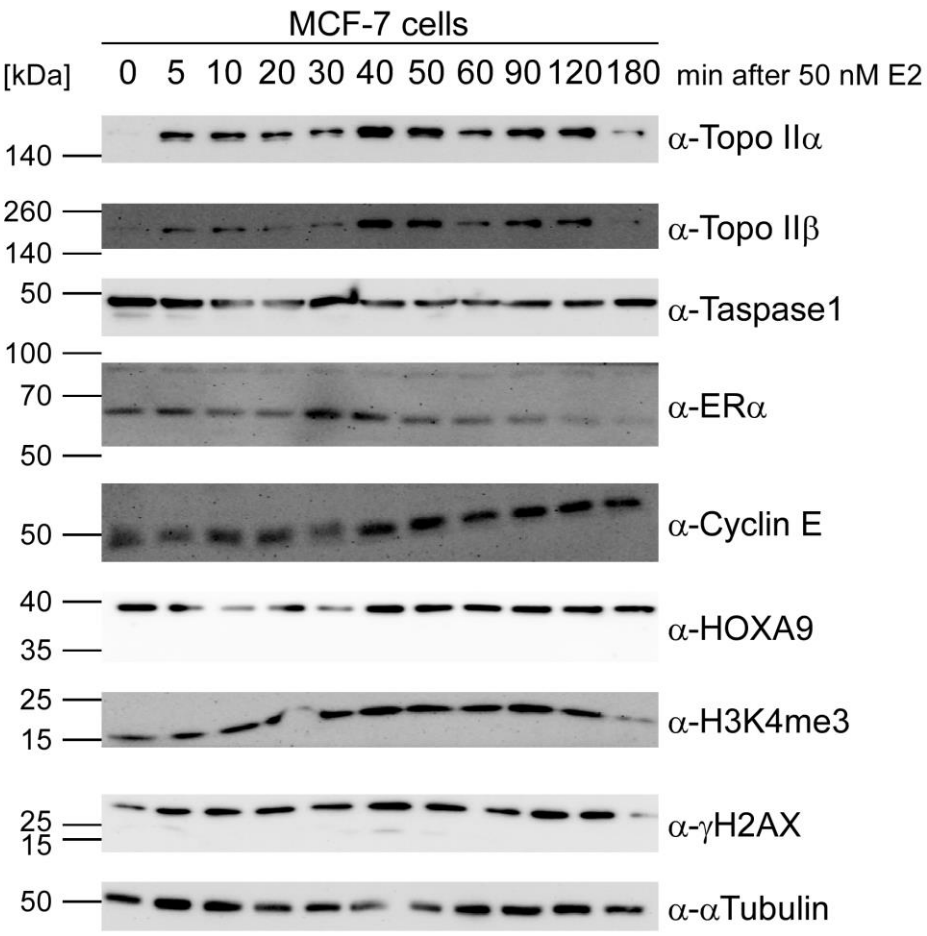

Figure S1. Estrogen response in MCF-7 cells. Protein expression level in MCF-7 cells after estrogen stimulation. RIPA cell lysates of MCF-7 cells were prepared at defined time points after 17β-estradiol (E2) treatment (50 nM) and analyzed for differential protein expression via immunoblotting and incubation with indicated antibodies.

A

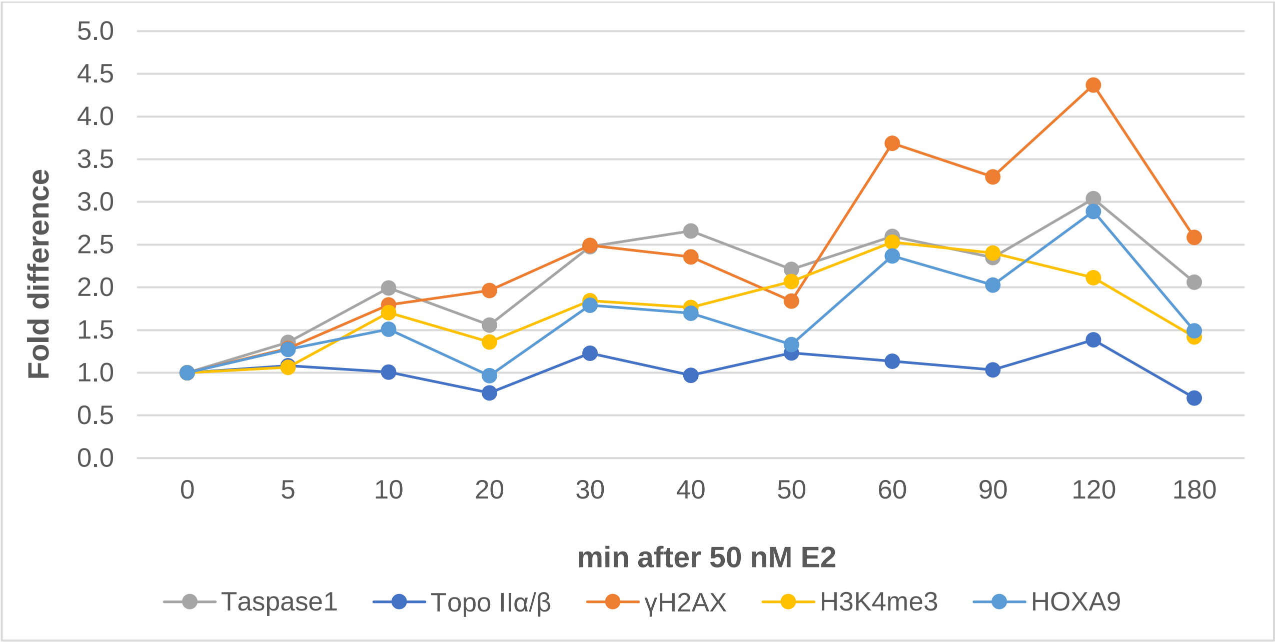

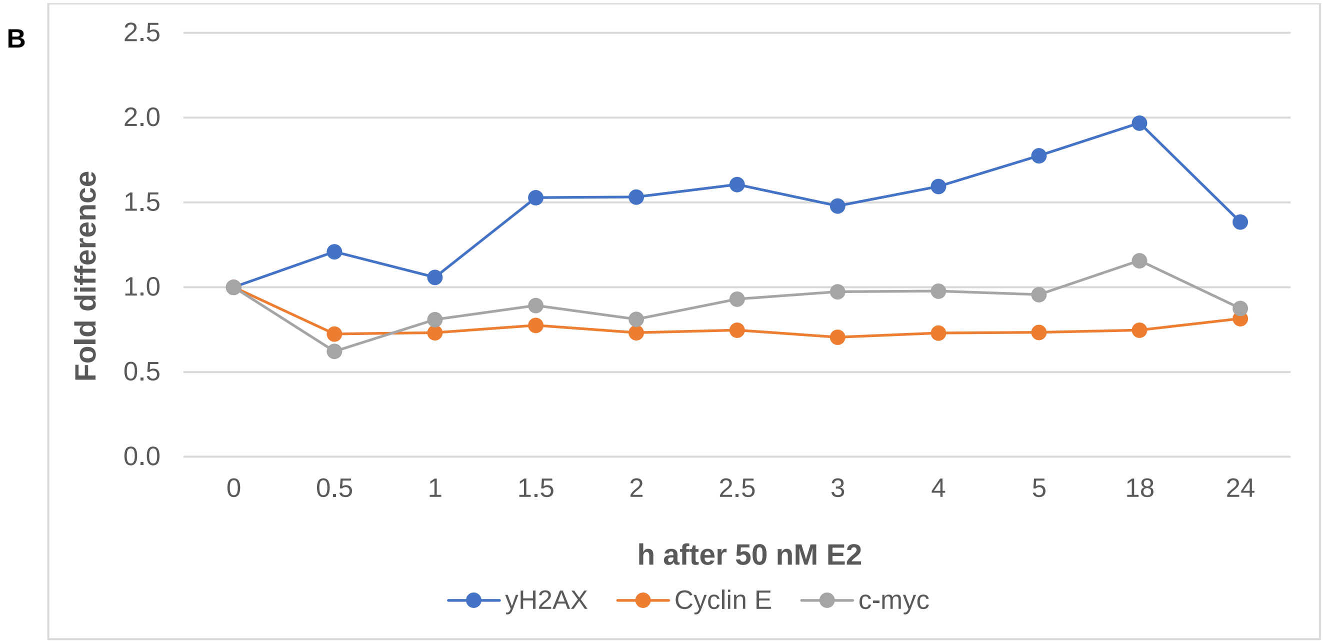

Figure S2. Densitometric quantification of E2 induction. The intensity of each band of the respective immunoblot of Figure 2A and Figure 2C were determined using Fiji. The amounts of the target protein were normalized to the loading control α-Tubulin. The relative target protein levels were compared across the samples. The quantification results of Figure 2A and Figure 2C immunoblots are depicted in (**A**) and (**B**), respectively.

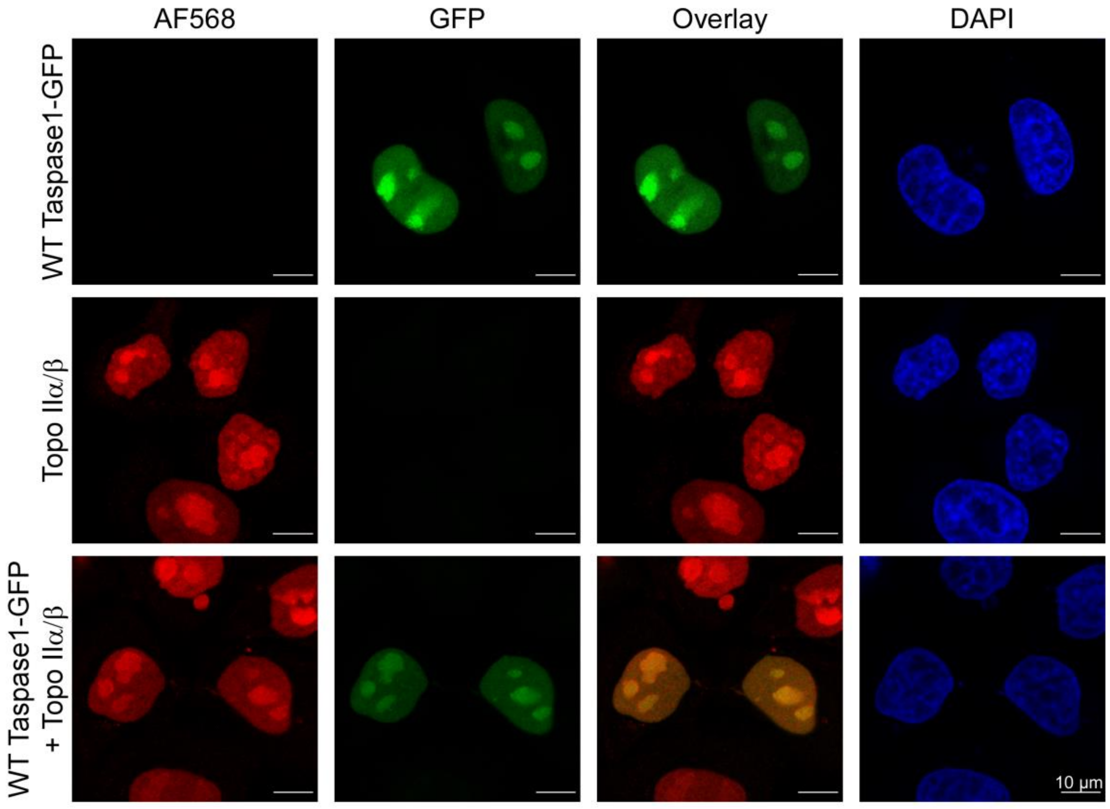

Figure S3. Taspase1 and Topo II co-localize in HeLa cells**.** Taspase1 and Topo II co-localize at the nuclei and nucleoli in HeLa cells. HeLa cells overexpressing WT Taspase1-GFP or untransfected cells were fixed and permeabilized and an immunofluorescence staining with a Topo IIα/β antibody and a secondary goat anti-rabbit IgG-AF568 antibody was performed. Cells were counterstained with DAPI and microscopically analyzed with the confocal laser scanning microscope SP8X Falcon (Leica Microsystems). Taspase1-GFP is depicted in green, Topo IIα/β in red and DNA in blue. Representative images are shown. Scale bar: 10 µm.

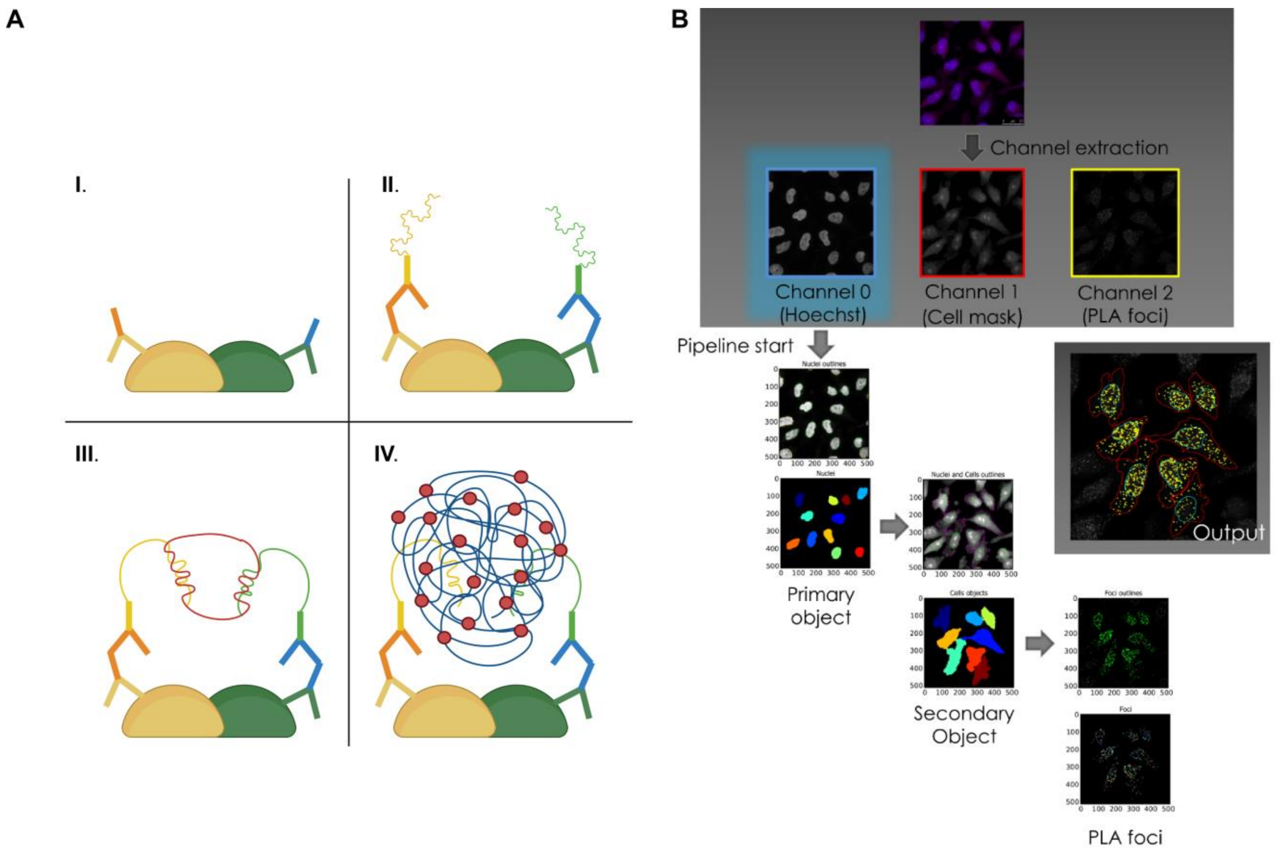

Figure S4. Schematic workflow of PLA procedure and image analysis pipeline. PLA procedure and image analysis by Cell Profiler. (A) The proteins of interest are recognized by their specific primary antibodies (I.). Secondary antibodies conjugated with oligonucleotides (PLA probes) bind the respective primary antibody (II.). If the target proteins interact with each other, they are in close proximity (<40 nm), thereby allowing the connector oligonucleotides to hybridize with both PLA probes and a closed circle DNA template is formed by ligation (III.). These circular structures are amplified using fluorescently labeled oligonucleotides to gain well-defined fluorescent dots (PLA foci) (IV.). This illustration was created with BioRender.com. (B) PLA foci were quantified with the cell image analysis software Cell Profiler 4.1.3. The Hoechst-stained nuclei were utilized to define the primary objects (blue) and the staining of the plasma membrane with HCS CellMask™ Deep Red for the definition of the secondary objects (red). Afterwards, PLA foci (yellow) were counted and assigned to the cellular compartments.

Table S1. Proteins identified in Taspase1-GFP complexes by mass spectrometry.

–LOG (P-value)	Difference	Protein names	Gene names
1,4533	1,4459	Protein AATF	AATF
1,3894	0,7370	ATP-binding cassette sub-family D member 3	ABCD3
2,2353	2,5468	Activator of basal transcription 1	ABT1
2,2388	2,4690	Protein ELYS	AHCTF1
2,5335	3,0525	ATPase family AAA domain-containing protein 2	ATAD2
3,2639	1,9490	Bromodomain adjacent to zinc finger domain protein 1A	BAZ1A
1,5911	1,0452	Tyrosine-protein kinase BAZ1B	BAZ1B
3,5857	2,5714	Bromodomain adjacent to zinc finger domain protein 2A	BAZ2A
1,7551	1,4528	Bloom syndrome protein	BLM
2,3170	2,2029	Ribosome biogenesis protein BMS1 homolog	BMS1
2,4213	1,6777	Ribosome biogenesis protein BOP1	BOP1
2,2828	1,8470	Nucleosome-remodeling factor subunit BPTF	BPTF
3,1971	5,7335	Ribosome biogenesis protein BRX1 homolog	BRIX1
2,2387	1,7486	Bystin	BYSL
1,7886	1,6806	Leydig cell tumor 10 kDa protein homolog	C19orf53
2,0007	1,4325	Uncharacterized protein C7orf50	C7orf50
1,5284	0,8471	Chromobox protein homolog 3	CBX3
2,7491	2,9865	Chromobox protein homolog 5	CBX5
2,9865	1,8999	Cell division cycle and apoptosis regulator protein 1	CCAR1
2,3920	1,8936	Coiled-coil domain-containing protein 137	CCDC137
3,2868	3,1116	Coiled-coil domain-containing protein 86	CCDC86
2,9838	1,2425	Coiled-coil domain-containing protein 94	CCDC94
2,8792	2,3459	Tumor suppressor ARF	CDKN2A
3,1602	5,2704	CCAAT/enhancer-binding protein zeta	CEBPZ
3,4640	3,3583	Centromere protein V	CENPV
4,0012	1,6876	Chromatin assembly factor 1 subunit A	CHAF1A
1,6820	1,2356	Charged multivesicular body protein 4b	CHMP4B
2,7958	2,3254	Coilin	COIL
1,2392	1,2645	Transcriptional repressor CTCF	CTCF
1,5579	2,5362	Spliceosome-associated protein CWC15 homolog	CWC15
1,6575	2,3532	DDB1- and CUL4-associated factor 13	DCAF13
3,6214	4,1330	Probable ATP-dependent RNA helicase DDX10	DDX10
1,8640	1,1750	Probable ATP-dependent RNA helicase DDX17	DDX17
3,8437	2,3457	ATP-dependent RNA helicase DDX18	DDX18
3,2941	1,9227	Nucleolar RNA helicase 2	DDX21
2,2878	2,2877	ATP-dependent RNA helicase DDX24	DDX24
3,8272	5,7230	Probable ATP-dependent RNA helicase DDX27	DDX27
2,3555	2,3129	Probable ATP-dependent RNA helicase DDX31	DDX31
3,8412	2,2557	ATP-dependent RNA helicase DDX50	DDX50
2,6405	2,1053	ATP-dependent RNA helicase DDX51	DDX51
2,5235	1,3294	Probable ATP-dependent RNA helicase DDX52	DDX52
2,8915	4,0868	ATP-dependent RNA helicase DDX54	DDX54

2,4602	2,6282	Probable ATP-dependent RNA helicase DDX56	DDX56
2,9556	1,6690	Putative ATP-dependent RNA helicase DHX30	DHX30
1,4653	1,7830	Putative ATP-dependent RNA helicase DHX33	DHX33
1,4248	1,9336	Probable ATP-dependent RNA helicase DHX37	DHX37
1,7726	2,4872	DnaJ homolog subfamily C member 9	DNAJC9
2,9996	3,4663	Deoxynucleotidyltransferase terminal-interacting protein 2	DNTTIP2
2,1280	1,0841	Protein dpy-30 homolog	DPY30
4,8000	5,0413	Probable rRNA-processing protein EBP2	EBNA1BP2
1,9080	1,2859	Polycomb protein EED	EED
1,6631	1,4313	Histone-lysine N-methyltransferase EHMT1	EHMT1
2,8442	1,7448	Histone-lysine N-methyltransferase EHMT2	EHMT2
2,3417	1,6139	Eukaryotic translation initiation factor 6	EIF6
1,6847	1,6106	Emerin	EMD
2,0667	0,9847	Ribosomal RNA small subunit methyltransferase NEP1	EMG1
2,2262	3,3614	ESF1 homolog	ESF1
3,1632	2,4612	Exosome component 10	EXOSC10
2,9519	3,6399	Exosome complex component RRP4	EXOSC2
1,6117	2,1803	Exosome complex component RRP40	EXOSC3
1,9324	2,6287	Exosome complex component RRP41	EXOSC4
3,7153	2,4533	Exosome complex component MTR3	EXOSC6
1,9823	2,4380	Exosome complex component RRP42	EXOSC7
2,6942	2,5019	40S ribosomal protein S30	FAU
3,2244	4,7385	rRNA 2-O-methyltransferase fibrillarin	FBL
4,8024	4,8114	pre-rRNA processing protein FTSJ3	FTSJ3
2,2796	2,4053	RNA-binding protein FUS	FUS
1,7476	1,2681	Fragile X mental retardation syndrome-related protein 2	FXR2
2,3209	2,5170	Transcriptional repressor p66-alpha	GATAD2A
5,3776	5,7870	Nucleolar GTP-binding protein 2	GNL2
3,1080	4,4706	Guanine nucleotide-binding protein-like 3	GNL3
2,2115	0,7465	G patch domain-containing protein 4	GPATCH4
3,7024	1,5834	Glutamate-rich WD repeat-containing protein 1	GRWD1
1,6823	1,6750	General transcription factor 3C polypeptide 1	GTF3C1
1,8963	2,1141	General transcription factor 3C polypeptide 2	GTF3C2
3,1262	2,7694	Nucleolar GTP-binding protein 1	GTPBP4
1,4253	1,2334	Histone H1x	H1FX
2,3602	1,2364	Core histone macro-H2A.2;Histone H2A	H2AFY2
1,3256	3,6191	Histone H3;Histone H3.3	H3F3B;H3F3A
2,5558	3,2446	High mobility group protein HMG-I/HMG-Y	HMGA1
1,8784	1,3071	High mobility group protein HMGI-C	HMGA2
1,2209	1,3666	Non-histone chromosomal protein HMG-14	HMGN1
1,6388	1,4234	Heterogeneous nuclear ribonucleoprotein A0	HNRNPA0
2,5787	1,5488	Heterogeneous nuclear ribonucleoprotein A1	HNRNPA1
3,7621	1,8814	Heterogeneous nuclear ribonucleoproteins A2/B1	HNRNPA2B1

3,0915	1,6334	Heterogeneous nuclear ribonucleoprotein A3	HNRNPA3
4,3729	3,1751		HNRNPDL
1,4516	1,2002	Heterogeneous nuclear ribonucleoprotein H3	HNRNPH3
1,9890	0,5093	Heterogeneous nuclear ribonucleoprotein L	HNRNPL
6,3544	2,3546	Heterogeneous nuclear ribonucleoprotein R	HNRNPR
1,3737	0,8358	Heterogeneous nuclear ribonucleoprotein U	HNRNPU
3,1137	1,8892	Heterogeneous nuclear ribonucleoprotein U-like protein 1	HNRNPUL1
1,7421	2,1135	Protein Red	IK
2,9789	1,0759	Interleukin enhancer-binding factor 3	ILF3
1,3468	1,0357	U3 small nucleolar ribonucleoprotein protein IMP4	IMP4
2,0401	1,4577	Inner centromere protein	INCENP
1,7065	2,1915	Interferon-stimulated 20 kDa exonuclease-like 2	ISG20L2
3,8081	2,6055	KH domain-containing, RNA-binding, signal transduction-associated protein 1	KHDRBS1
3,0654	5,1239	Pumilio domain-containing protein KIAA0020	KIAA0020
2,6767	2,3267	Protein virilizer homolog	KIAA1429
2,5467	1,1927	Kinesin-like protein KIF18B	KIF18B
2,1445	2,0079	Protein KRI1 homolog	KRI1
2,6682	3,4815	KRR1 small subunit processome component homolog	KRR1
3,1549	1,5708	La-related protein 7	LARP7
3,3361	2,1252	Ribosomal biogenesis protein LAS1L	LAS1L
2,4959	2,3177	DNA ligase 3	LIG3
1,9398	1,3669	Protein lin-28 homolog B	LIN28B
2,2276	2,4426	Cell growth-regulating nucleolar protein	LYAR
3,0941	3,5466	Protein MAK16 homolog	MAK16
6,0013	5,0391	Mediator of DNA damage checkpoint protein 1	MDC1
2,1020	1,8919	Midasin	MDN1
3,7258	2,8227	Methyl-CpG-binding protein 2	MECP2
1,5049	0,7477	Mediator of RNA polymerase II transcription subunit 15	MED15
1,5297	1,0902	Mediator of RNA polymerase II transcription subunit 6	MED6
2,2304	3,6387	Antigen KI-67	MKI67
1,6823	1,8192	U3 small nucleolar ribonucleoprotein protein MPP10	MPHOSPH10
1,4856	1,2393	Myosin phosphatase Rho-interacting protein	MPRIP
1,4130	2,3333	mRNA turnover protein 4 homolog	MRTO4
1,6398	1,5918	DNA mismatch repair protein Msh2	MSH2
1,3441	0,9219	DNA mismatch repair protein Msh6	MSH6
1,7886	0,7443	Metastasis-associated protein MTA2	MTA2
2,4980	1,3658	Metal-response element-binding transcription factor 2	MTF2
3,4682	5,1108	Myb-binding protein 1A	MYBBP1A
2,3668	1,5688	Myosin-10	MYH10
1,9810	1,6852	Myosin-9	MYH9
2,0480	0,7459	Unconventional myosin-Ib	MYO1B
1,4272	4,6683	Myelin transcription factor 1-like protein	MYT1L
3,1142	0,7509	N-acetyltransferase 10	NAT10
2,9173	**3,6937**	**Nucleolin**	**NCL**

2,6296	1,1920	Neuroguidin	NGDN
3,3918	4,5364	MKI67 FHA domain-interacting nucleolar phosphoprotein	NIFK
2,3136	1,2635	60S ribosome subunit biogenesis protein NIP7 homolog	NIP7
1,2493	1,9966	NF-kappa-B-activating protein;NKAP-like protein	NKAP;NKAPL
1,3659	2,1529	Notchless protein homolog 1	NLE1
2,2462	0,9134	Nicotinamide/nicotinic acid mononucleotide adenylyltransferase 1	NMNAT1
2,1969	3,5726	Nucleolar complex protein 2 homolog	NOC2L
3,5683	2,4030	Nucleolar complex protein 3 homolog	NOC3L
2,1677	1,3241	Nucleolar complex protein 4 homolog	NOC4L
1,8872	2,7030	Nucleolar protein 10	NOL10
2,3551	1,0486	Nucleolar protein 11	NOL11
1,9948	1,1984	Nucleolar protein 6	NOL6
1,8760	2,8836	Nucleolar protein 7	NOL7
2,3551	2,3132	Nucleolar protein 8	NOL8
1,8825	2,4925	Polynucleotide 5-hydroxyl-kinase NOL9	NOL9
1,9142	2,9375	Nucleolar and coiled-body phosphoprotein 1	NOLC1
2,7865	4,0995	Non-POU domain-containing octamer-binding protein	NONO
2,9998	4,6736	Nucleolar protein 14	NOP14
3,2568	1,5258	Nucleolar protein 16	NOP16
4,0739	5,8043	Probable 28S rRNA (cytosine(4447)-C(5))-methyltransferase	NOP2
2,4831	3,0370	Nucleolar protein 56	NOP56
3,2937	**2,0602**	**Nucleophosmin**	**NPM1**
2,1155	1,4867	Nucleoplasmin-3	NPM3
2,6281	3,3155	Ribosome biogenesis protein NSA2 homolog	NSA2
1,8647	1,4105	E3 SUMO-protein ligase NSE2	NSMCE2
3,0715	2,4545	Nuclear mitotic apparatus protein 1	NUMA1
2,0293	1,2580	Nuclear valosin-containing protein-like	NVL
2,0972	2,8510	p21-activated protein kinase-interacting protein 1	PAK1IP1
3,2052	0,8923	Poly [ADP-ribose] polymerase 1	PARP1
1,7778	1,8535	Protein polybromo-1	PBRM1
2,0428	3,8468	Protein RRP5 homolog	PDCD11
3,1208	2,1211	Proline-, glutamic acid- and leucine-rich protein 1	PELP1
2,9570	1,1176	Pescadillo homolog	PES1
3,1393	1,1552	Prohibitin-2	PHB2
2,0600	0,8946	Histone lysine demethylase PHF8	PHF8
2,4179	3,2203	PH-interacting protein	PHIP
2,3744	2,0860	Ribonucleases P/MRP protein subunit POP1	POP1
4,9385	5,1363	Suppressor of SWI4 1 homolog	PPAN-P2RY11
1,4190	0,9349	Peptidyl-prolyl cis-trans isomerase A	PPIA
1,3919	2,2918	Serine/threonine-protein phosphatase PP1-beta catalytic subunit	PPP1CB
2,7391	0,6762	DNA-dependent protein kinase catalytic subunit	PRKDC
3,0218	2,8645	PC4 and SFRS1-interacting protein	PSIP1
3,7072	1,8511	Periodic tryptophan protein 1 homolog	PWP1
2,3078	1,6087	Periodic tryptophan protein 2 homolog	PWP2
1,2198	2,2100	GTP-binding nuclear protein Ran	RAN
1,4571	1,4034	Histone-binding protein RBBP4	RBBP4

2,2588	3,8934	RNA-binding protein 14	RBM14
1,9925	1,4997	Probable RNA-binding protein 19	RBM19
4,6944	2,0638	RNA-binding protein 28	RBM28
3,8715	4,6912	RNA-binding protein 34	RBM34
1,4482	1,8701	RNA-binding protein 8A	RBM8A
2,2848	3,0370	RNA-binding motif protein, X chromosome	RBMX
3,1362	2,7533	Regulator of chromosome condensation	RCC1
2,2262	1,6531	RNA exonuclease 4	REXO4
1,5457	0,9420	Replication factor C subunit 1	RFC1
1,2174	1,7923	Replication factor C subunit 2	RFC2
2,7269	1,1018	Telomere-associated protein RIF1	RIF1
2,6765	3,8658	Ribosome production factor 1	RPF1
2,7714	3,1937	Ribosome production factor 2 homolog	RPF2
2,6137	3,1016	60S ribosomal protein L10	RPL10
2,5289	1,0385	60S ribosomal protein L11	RPL11
2,4151	1,7471	60S ribosomal protein L13	RPL13
2,6098	3,8113	60S ribosomal protein L13a	RPL13A
2,7674	2,2360	60S ribosomal protein L14	RPL14
2,0396	3,2779	60S ribosomal protein L15	RPL15
3,5313	1,8920	60S ribosomal protein L18	RPL18
2,7361	3,8088	60S ribosomal protein L18a	RPL18A
2,8662	3,5085	60S ribosomal protein L21	RPL21
4,0759	5,4227	60S ribosomal protein L23a	RPL23A
2,2629	3,5709	60S ribosomal protein L26	RPL26
3,7918	2,1390	60S ribosomal protein L27	RPL27
4,8333	5,7166	60S ribosomal protein L27a	RPL27A
2,3571	1,6852	60S ribosomal protein L28	RPL28
3,4481	2,6418	60S ribosomal protein L3	RPL3
5,1083	4,7525	60S ribosomal protein L32	RPL32
1,9921	3,1082	60S ribosomal protein L34	RPL34
2,9679	2,7037	60S ribosomal protein L35a	RPL35A
2,4334	4,4540	60S ribosomal protein L36	RPL36
2,5703	2,3391	60S ribosomal protein L4	RPL4
2,9956	2,9257	60S ribosomal protein L5	RPL5
2,5501	3,6173	60S ribosomal protein L6	RPL6
3,3193	1,8135	60S ribosomal protein L7	RPL7
3,4192	2,6476	60S ribosomal protein L7a	RPL7A
1,4624	1,0244	60S ribosomal protein L7-like 1	RPL7L1
2,8245	3,2000	60S ribosomal protein L8	RPL8
3,3734	3,3316	60S acidic ribosomal protein P1	RPLP1
3,4208	1,8580	60S acidic ribosomal protein P2	RPLP2
2,2949	1,6955	Ribonuclease P protein subunit p30	RPP30
1,2985	2,0673	40S ribosomal protein S11	RPS11
2,7561	1,1397	40S ribosomal protein S14	RPS14
1,8700	1,7803	Active regulator of SIRT1	RPS19BP1
1,9480	0,8109	40S ribosomal protein S23	RPS23
1,8627	1,8852	40S ribosomal protein S24	RPS24
2,0512	1,9780	40S ribosomal protein S3a	RPS3A
2,2152	3,7698	40S ribosomal protein S6	RPS6
2,3653	2,2779	40S ribosomal protein S8	RPS8
2,7359	3,0458	Ribosomal RNA processing protein 1 homolog A	RRP1
3,1184	2,8782	RRP12-like protein	RRP12
2,9985	2,8537	RRP15-like protein	RRP15
4,1806	5,3675	Ribosomal RNA processing protein 1 homolog B	RRP1B
2,3326	1,7248	Ribosomal RNA processing protein 36 homolog	RRP36

1,9134	1,3803	Ribosomal RNA-processing protein 7 homolog A	RRP7A
2,3450	2,6344	Ribosomal RNA-processing protein 8	RRP8
1,9295	3,1653	U3 small nucleolar RNA-interacting protein 2	RRP9
2,6724	2,5263	Ribosome biogenesis regulatory protein homolog	RRS1
2,0376	2,2352	Round spermatid basic protein 1-like protein	RSBN1L
1,3579	1,5472	Remodeling and spacing factor 1	RSF1
2,9922	4,6573	Ribosomal L1 domain-containing protein 1	RSL1D1
1,5620	3,0653	Probable ribosome biogenesis protein RLP24	RSL24D1
1,3345	0,9517	tRNA-splicing ligase RtcB homolog	RTCB
4,9193	1,6924	Scaffold attachment factor B1	SAFB
1,4793	1,2945	U4/U6.U5 tri-snRNP-associated protein 1	SART1
1,8119	2,7092	Sentrin-specific protease 3	SENP3
1,7933	0,9076	Plasminogen activator inhibitor 1 RNA-binding protein	SERBP1
2,8286	1,3863	Splicing factor 3B subunit 2	SF3B2
1,5210	2,5622	Splicing factor 3B subunit 4	SF3B4
1,3828	0,9175	Transcription activator BRG1	SMARCA4
2,0230	0,6770	SWI/SNF complex subunit SMARCC1	SMARCC1
1,4402	1,2264	Structural maintenance of chromosomes protein 1A	SMC1A
2,9005	1,0818	Structural maintenance of chromosomes protein 5	SMC5
1,7436	2,1568	Structural maintenance of chromosomes flexible hinge domain-containing protein 1	SMCHD1
2,3830	2,8455	SNW domain-containing protein 1	SNW1
2,5907	1,7429	Spermatogenesis-associated protein 5	SPATA5
2,5672	2,2388	Msx2-interacting protein	SPEN
2,4393	1,5938	Serum response factor-binding protein 1	SRFBP1
1,6406	1,9874	Serine/arginine-rich splicing factor 9	SRSF9
1,4230	1,1621	Lupus La protein	SSB
3,2840	1,9000	FACT complex subunit SSRP1	SSRP1
2,7542	2,2523	Double-stranded RNA-binding protein Staufen homolog 1	STAU1
1,6098	0,9703	SURP and G-patch domain-containing protein 2	SUGP2
1,8460	1,4655	FACT complex subunit SPT16	SUPT16H
1,3450	1,6434	Heterogeneous nuclear ribonucleoprotein Q	SYNCRIP
2,6713	2,7237	TATA-binding protein-associated factor 2N	TAF15
7,3129	**10,4432**	**Threonine aspartase 1**	**TASP1**
2,2609	0,9266	Transducin beta-like protein 3	TBL3
1,7632	1,1928	Transcription elongation regulator 1	TCERG1
2,3202	1,4735	Testis-expressed sequence 10 protein	TEX10
3,9787	2,1365	Transcription factor A, mitochondrial	TFAM
3,1735	1,2028	Tight junction protein ZO-1	TJP1
1,5872	0,9450	Target of EGR1 protein 1	TOE1
3,4893	**2,1030**	**DNA topoisomerase 2-alpha**	**TOP2A**
1,9182	**3,3554**	**DNA topoisomerase 2-beta**	**TOP2B**
2,0187	0,9680	Tumor suppressor p53-binding protein 1	TP53BP1
2,7486	1,1862	TRMT1-like protein	TRMT1L
1,5322	2,2831	Nucleolar transcription factor 1	UBTF
6,8028	4,4660	Ubiquitin carboxyl-terminal hydrolase 36	USP36
3,1106	2,1976	Probable U3 small nucleolar RNA-associated protein 11	UTP11L

2,8875	2,3968	U3 small nucleolar RNA-associated protein 14 homolog A	UTP14A
1,8621	2,2077	U3 small nucleolar RNA-associated protein 15 homolog	UTP15
1,9902	3,4326	U3 small nucleolar RNA-associated protein 18 homolog	UTP18
2,6422	1,6148	Small subunit processome component 20 homolog	UTP20
2,7767	2,6975	Something about silencing protein 10	UTP3
2,2812	1,3586	U3 small nucleolar RNA-associated protein 6 homolog	UTP6
1,8716	2,4485	WW domain-binding protein 11	WBP11
3,0287	1,3139	Ribosome biogenesis protein WDR12	WDR12
2,8195	3,2388	WD repeat-containing protein 18	WDR18
3,4648	1,2625	WD repeat-containing protein 3	WDR3
2,9750	2,4686	pre-mRNA 3 end processing protein WDR33	WDR33
1,9633	0,7948	WD repeat-containing protein 36	WDR36
5,0338	1,0301	WD repeat-containing protein 43	WDR43
3,3921	2,6212	WD repeat-containing protein 46	WDR46
1,3990	1,0967	WD repeat-containing protein 5	WDR5
2,6216	1,2233	WD repeat-containing protein 74	WDR74
4,0020	0,8301	WD repeat-containing protein 76	WDR76
1,2599	1,0713	Protein Wiz	WIZ
2,3551	1,4047	DNA repair protein XRCC1	XRCC1
1,6563	0,8002	Nuclease-sensitive element-binding protein 1	YBX1
2,9222	1,4798	YTH domain-containing protein 1	YTHDC1
1,6568	1,5233	Zinc finger CCCH-type antiviral protein 1	ZC3HAV1
3,3099	2,5689	Zinc finger RNA-binding protein	ZFR
2,0564	1,6639	Zinc finger MYM-type protein 4	ZMYM4
1,6835	1,7883	Zinc finger protein 280C	ZNF280C
2,6580	2,7459	Zinc finger protein 638	ZNF638
